# Does Conflict of Interest Disclosure Worsen Bias?

**DOI:** 10.1371/journal.pmed.1001210

**Published:** 2012-04-24

**Authors:** The PLoS Medicine Editors

## Abstract

The *PLoS Medicine* Editors discuss financial conflicts of interest at the American Psychiatric Association, and raise concerns about new evidence from the social sciences that suggests conflict of interest disclosures worsen rather than ameliorate bias.

On March 13, 2012 *PLoS Medicine* published an analysis by Lisa Cosgrove and Sheldon Krimsky [1] that examined the financial conflicts of interest of members of the American Psychiatric Association (APA) responsible for updating the Diagnostic and Statistical Manual of Mental Disorders (DSM), the so-called bible of psychiatry. Despite a new APA policy designed to address conflicts of interest (COIs), nearly 70% of current DSM-5 task force members have financial relationships with pharmaceutical companies, up from 57% for the manual's previous version. 83% of current contributors to the psychotic disorders section, and everyone responsible for the sleep disorder section, have links to the pharmaceutical industry. Wide media coverage and commentary about these findings [2]–[5] have raised concerns that so many of the experts charged with the responsibility of defining mental health conditions and treatments have financial ties to the very companies that sell drug treatments for mental health. It is widely established that financial conflicts of interest impair objectivity and integrity in medicine.

Concerns about the conflicts of interests associated with the APA—undeniably the leading authority for psychiatry and mental health—are critical, not least because of the association's legacy of involvement with the pharmaceutical industry: the psychiatric profession receives more money than any other medical specialty [6] and has recently been scandalized by cases of ghostwriting and publication bias. Their judgments define mental illness, thus legitimizing some disorders and denying others, and determine what warrants treatment and how. The DSM is used by insurance companies, hospitals, courts, prisons, schools, researchers, regulators, and government agencies to define who is sick/abnormal and who is not. The expansion of diagnostic categories and new diagnoses (and thus markets) in every DSM is said to be a virtual “bonanza for the pharmaceutical industry” [9]. And on the other side of the coin, the DSM is a boon for the APA, which sold over a million copies of the DSM-IV; 20% of APA funding is said to now come from pharmaceutical companies [8].

Cosgrove and Krimsky also identified several worrying gaps in the APA's new COI policy (previous DSMs in 1952, 1968, and 1980 were not subject to COI policies). While the policy limits the amount panel members can receive from drug companies annually to US$10,000 and of their company stock holdings to US$50,000, these are still considerable amounts. (Even small gifts invoke obligations to reciprocate [6]). Worse, the policy does not consider unrestricted research grants from pharmaceutical companies to be problematic and does not require they be disclosed. Participation in lucrative speakers' bureaus (networks of prominent physicians designed to influence communities of prescribers and usually forbidden in medical schools) is likewise permitted under the APA's policy, and the monies received for participation are required only to be reported as honoraria, thus concealing their true genesis. The APA has responded to the *PLoS Medicine* analysis by saying that the DSM-5 development process “is the most open and transparent of any previous edition of the DSM” [2].

But are disclosure mandates simply a band-aid on a unrelenting problem of bias?

Disclosure is generally considered preferable to nondisclosure, because it makes explicit and transparent details that are important to the interpretation, credibility, and value of the information presented—vital in the context of clinical decision-making and patient care. But the overemphasis and reliance on disclosure policies is exactly what leaves the real problem of the conflict of interest unaddressed.

Disclosure has severe limits as a strategy for mitigating bias. Cosgrove and Krimsky mention three reasons: that disclosure alone merely shifts “secret bias” to “open bias”; that it sometimes involves so much information about ties to the industry, for example, that the reader is blinded by the sheer “signal to noise ratio”; and that disclosure may be perceived as absolving a person from their responsibility for managing their conflict [1].

Even more compelling is evidence emerging from the social sciences that suggests disclosure to be not only ineffective but also regressive. Decision scientist George Loewenstein and colleagues have argued that disclosure can actually lead doctors to give biased advice, either through strategic exaggeration (whereby more biased advice is provided to counteract anticipated discounting), or “moral licensing” such that advice is legitimized because advisees “have been warned” (that is, caveat emptor or “buyer beware”) [7]. Their experiments have essentially shown that bias is considerably greater when conflicts of interest are disclosed. Worse, because Loewenstein and colleagues have demonstrated that advisees (i.e., patients) both think that their advisers (i.e., doctors) would never intentionally mislead them and tend not to discount advice in light of conflicts, disclosure policies will never be the solution and are very likely exacerbating the problem of bias in medicine [7].

Extending this analysis to the APA's DSM, the result would be disastrous if the public or physicians were to disregard their concerns about financial conflicts of interest in deference to the authority of the APA. And if clinical experts were to believe that disclosure alone made them impervious to bias, their advice forming the DSM may be even more favorable toward the pharmacological products and markets their industry funders seek and uphold.

Indeed, if disclosure worsens bias, then this is a game-changer for discussion and debate about managing conflicts of interest in medicine. Journals, professional associations, clinical guideline developers, and others need to worry not just that disclosure provides a band-aid to the real problem of the COI itself, but that any attempt to stem the trouble through disclosure policies may actually be worsening the problem.

## Abbreviations

APA: American Psychiatric Association
COI: conflict of interest
DSM: Diagnostic and Statistical Manual of Mental Disorders
